# Severe Obesity in Women Can Lead to Worse Memory Function and Iron Dyshomeostasis Compared to Lower Grade Obesity

**DOI:** 10.1155/2023/7625720

**Published:** 2023-04-17

**Authors:** Jessica M. V. Pino, Vitória F. Silva, Marcos Mônico-Neto, Danielle C. Seva, Melissa Y. Kato, July N. Alves, Gabriela C. Pereira, Hanna Karen M. Antunes, Thales D. Galvao, Lia R. A. Bitterncourt, Sergio Tufik, Lysien I. Zambrano, Ana R. Dâmaso, Lila M. Oyama, David Thivel, Raquel M. S. Campos, Kil S. Lee

**Affiliations:** ^1^Department of Biochemistry, Universidade Federal de São Paulo, Sao Paulo, Brazil; ^2^Post Graduated Program of Interdisciplinary Health Sciences, Universidade Federal de São Paulo, Santos, Brazil; ^3^BariMais Clinic-Integrated Medicine, Sao Paulo, Brazil; ^4^Department of Psychobiology, Universidade Federal de São Paulo, Sao Paulo, Brazil; ^5^Institute for Research in Medical Sciences and Right to Health (ICIMEDES)/Scientific Research Unit (UIC), Faculty of Medical Sciences (FCM), National Autonomous University of Honduras (UNAH). Tegucigalpa, Honduras, Honduras; ^6^Post Graduate Program of Nutrition, Universidade Federal de São Paulo, Sao Paulo, Brazil; ^7^Clermont Auvergne University, EA 3533, Laboratory of the Metabolic Adaptations to Exercise under Physiological and Pathological Conditions (AME2P), CRNH-Auvergne, Clermont-Ferrand, France

## Abstract

**Objective:**

Obesity is one of the modifiable risk factors for dementia. Insulin resistance, the abundance of advanced glycated end-products, and inflammation are some of the mechanisms associated with the lower cognitive performance observed in obesity. This study aims to evaluate the cognitive function of subjects with distinct degrees of obesity, comparing class I and II obesity (OBI/II) to class III obesity (OBIII), and to investigate metabolic markers that can distinguish OBIII from OBI/II. *Study Design.* This is a cross-sectional study, in which 45 females with BMI varying from 32.8 to 51.9 kg/m^2^ completed a set of 4 cognitive tests (verbal paired-associate test, stroop color, digit span, and Toulouse–Pieron cancellation test) and their plasma metabolites, enzymes, and hormones related to glycemia, dyslipidemia, and liver function, as well as the biomarkers of iron status, were concomitantly analyzed.

**Results:**

OBIII showed lower scores in the verbal paired-associate test compared to OBI/II. In other cognitive tests, both groups showed similar performance. OBIII presented a lower iron status compared to OBI/II based on total iron binding capacity, degree of transferrin saturation, hemoglobin, mean corpuscular volume, and mean corpuscular hemoglobin. The levels of indicators for glycemia, liver function, and lipid metabolism were similar in both groups. Analysis of plasma metabolites showed that OBIII had lower levels of pyroglutamic acid, myoinositol, and aspartic acid and higher levels of D-ribose than OBI/II.

**Conclusion:**

Iron is an essential micronutrient for several metabolic pathways. Thus, iron dyshomeostasis observed in severe obesity may aggravate the cognitive impairment by altering metabolic homeostasis and enhancing oxidative stress. These findings can contribute to searching for biomarkers that indicate cognitive performance in the population with obesity.

## 1. Introduction

Obesity is a complex pathological condition, which is mainly characterized by excessive amounts of body fat. Its etiology is often associated with inadequate diet and sedentary behavior, but other factors such as genetic background, epigenetic factors, socioeconomic status, and mood disorders can also promote obesity [1]. Currently, more than 13% of the worldwide adult population suffers from obesity, and a trend of increasing prevalence has been reported [1, 2].

Adipose tissues are important energy reservoirs, but also play a role as an endocrine organ that produces adipokines, which regulate food intake, energy expenditure, and body fat deposition. For instance, leptin is released when fat depot or acute caloric intake are increased and activate the anorexigenic neural circuit [3], while adiponectin is induced by weight loss and regulates insulin sensitivity as well as glucose and lipid metabolism [4]. Thus, excessive amounts of body fat can impair energy homeostasis and lead to a life-threatening condition. A BMI above 40 kg/m^2^, for instance, has been shown to increase the risk of death by tenfold, then being classified as severe obesity or class III obesity [5], but it is often preceded by the development of other chronic comorbidities such as diabetes and cardiovascular diseases in earlier stages of obesity [6, 7].

Studies have reported that obesity developed in middle age can also increase the risk of Alzheimer disease in later stages of life [8]. Moreover, obesity has been associated with reduced white and grey matter of several brain areas [6]. However, the detailed mechanisms by which obesity affects brain anatomy and functions are not completely elucidated.

Metabolism and energy balance are closely related with brain health and cognitive function. Thus, it is plausible to think that altered circulating metabolites caused by obesity can change the set of nutrients available to the brain and consequently the way that the brain produces its energy [6]. In fact, chronic hyperglycemia, shown by elevated glycated hemoglobin or accumulated advanced glycation end-products, has been associated with poor cognitive performance [9–11]. Moreover, several groups showed that insulin resistance also significantly contributes to the impairment of brain function, including the development of Alzheimer's disease, which has been denominated as type 3 diabetes by a group of researchers [6, 12]. Certain fatty acids can also pass the blood brain barrier and activate the immune system and elicit inflammatory responses [13]. Saturated fatty acids can induce astrocytes to release tumor necrosis factor-*α* (TNF-*α*) and interleukin-6 (IL-6) through toll-like receptor 4 signaling, while unsaturated fatty acids have a protective role [14].

Obesity not only can alter the circulating metabolites but also can affect the integrity of the blood-brain barrier. It is well accepted that obesity is an inflammatory state, and adipose tissue can contribute to the systemic proinflammatory cytokine pools [6]. A previous study has reported that diet-induced obesity promoted systemic inflammation, oxidative stress, and blood-brain barrier disruption in an animal model, which was aggravated by aging [15].

Altogether, these findings indicate that altered circulating metabolites and inflammatory molecules can affect the physiology of the brain and cognitive functions. Thus, thorough investigation of the systemic changes associated with obesity can help to better understand the relationship between obesity and cognitive function. In this study, we investigated whether a higher degree of obesity can worsen performance in cognitive tests, and searched for potential metabolic markers that can negatively affect cognitive function.

## 2. Methods

### 2.1. Study Participants

The sample consisted of 45 women with their age ranging from 18 to 50 and BMI varying from 32.8 to 51.9 kg/m^2^. The recruitment of subjects was carried out between April 2019 and March 2020. The inclusion criteria were as follows: individuals with BMI >30 kg/m^2^ and with or without comorbidities (diabetes mellitus, systolic arterial hypertension, dyslipidemia, coronary artery disease, obstructive sleep apnea, asthma, debilitating arthritis, reflux disease gastroesophageal, and nonalcoholic hepatic steatosis). The exclusion criteria were as follows: chronic alcoholism, use of neuroleptic and hypnotic drugs, decompensated clinical illnesses, and psychiatric illnesses. Biochemical data and clinical history were extracted from medical records. All subjects signed the informed consent form, and the study was designed and conducted by following the guidelines of the Brazilian National Research Ethics committee (CONEP: Comissao Nacional de Etica em Pesquisa) and approved by the Ethics Committee of UNIFESP (number: 0765.0006.07/2018).

### 2.2. Body Composition

Blood samples were collected between 8:00 and 10:00 A.M. after an overnight fast (8−12 h approximately). Soon after the blood collection, anthropometric assessment was carried out with minimal clothing and no metallic accessories were allowed. The subjects were not allowed to perform physical exercise 24 hours prior to measurements. Body fat mass (BFM), BMI, visceral fat area/height (VFA), waist-to-hip ratio, fat free mass (FFM), and skeletal muscle mass (SMM) were obtained using bioelectrical impedance (InBody 720®, Seoul, South Korea). At the beginning of the evaluation, the name, age, and gender of the participants were collected.

### 2.3. Cognitive Tests

After the measurement of the body composition, breakfast was provided and then cognitive tests were applied. At this stage, the participants also disclosed their education level. All participants followed the same course of evaluation.

Cognitive function was assessed using 4 tests: verbal paired-associate test, digit span, Toulouse–Piéron cancellation test, and stroop color. The applications of the tests were always carried out in the same place, by the same evaluators, and in the same test order. The test battery lasted approximately 30 minutes. The verbal paired-associate test assesses associative memory. Initially, the evaluator verbally exposed 8 pairs of words, 4 with semantic association and 4 without semantic relationship, and then participants were asked to recall paired-associated word in response to prompted words from the evaluator. Each trial is scored and the sum of 3 trials was defined as the total recall. After 3 trials, one additional trial was carried out after 30 min, which was designated as delayed recall [16]. For the digit span test, the evaluator verbally presented sequences of numbers, and then participants were asked to repeat the sequence. In the second test, the evaluator also verbally presented a sequence of numbers, but the participants were asked to repeat the numbers in reverse order [17]. The Toulouse–Pieron cancellation test is a psychometric test designed to measure the reaction speed and accuracy in a simple perceptual task. Participants were asked to find and mark 4 different symbols previously presented in a box containing several symbols [18]. The stroop color cognitive test assesses the attention and executive function. In the first test, a card containing colored squares (green, blue, yellow, and red) was shown to participants who were required to speak the colors of the squares as quickly as possible. The second card contained random words printed with the same colors used in the first card, and participants were asked to speak the ink color regardless of the written word. The third card contained color names printed in conflicting-color using the same set of the first card, and the participants were requested to speak the ink color. Time and hits/misses are recorded for each card [19].

### 2.4. Serum Analysis

IL-6, hepcidin, adiponectin, leptin, and fibroblast growth factor-21 (FGF-21) were analyzed using the respective ELISA kits: IL-6 (DY206-05; R&D Systems, Minneapolis, MN), hepcidin (DY8307-05; R&D Systems, Minneapolis, MN), adiponectin (DY1065-05; R&D Systems, Minneapolis, MN), leptin (DY398-05; R & D Systems, Minneapolis, MN), and e FGF21 (DF2100; R & D Systems, Minneapolis, MN), according to the manufacturer's instruction.

### 2.5. Metabolic Profiling

**Figure 1:**
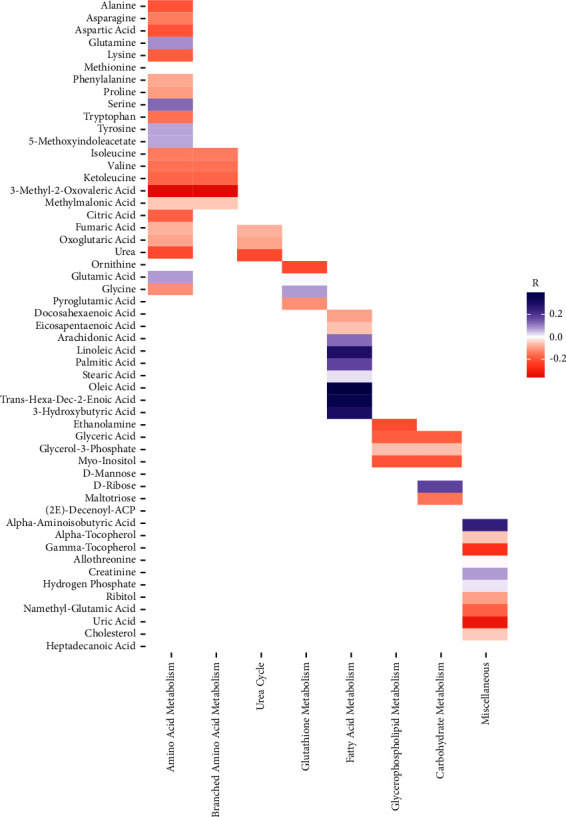
Correlation table. Abundance of each metabolite was defined based on mass spectral peaks. The table shows the correlation between body fat mass and abundance of 53 metabolites identified by an untargeted method.

Plasma samples were analyzed using gas chromatography coupled with a quadrupole mass spectrometer (GCMS-QP2020 NX, Shimadzu Co, Kyoto, Japan), as described elsewhere [20]. Instrument control, data acquisition, and data processing were performed using LabSolutions software (GCMS version 4.5, Shimadzu Co., Japan). The identification of metabolites was performed by comparing the spectra obtained from the analyzed samples with the reference spectra acquired under the same experimental conditions. After identification of the molecules by NIST and Smart Metabolite libraries, the data were exported to Excel (Microsoft Office) for statistical treatment. Fifty-three unique metabolites were identified, as shown in Figure 1. The univariate statistical analysis was performed in Metaboanalyst 5.0 (https://www.metaboanalyst.ca/).

Furthermore, the following biochemical data were extracted from the electronic medical record of the participants: serum iron, total iron binding capacity (TIBIC), transferrin saturation (TSAT), ferritin, hemoglobin, hematocrit, mean corpuscular volume (MCV), mean corpuscular hemoglobin (MCH), mean corpuscular hemoglobin concentration (MCHC), and red cell distribution width (RDW) as biomarkers of iron status; glycaemia, glycated hemoglobin, insulin, and homeostasis model assessment of insulin resistance (HOMA-IR) as variables related to glycemia; alanine aminotransferase, aspartate aminotransferase, gamma-glutamyltransferase, total bilirubin, direct bilirubin, and indirect bilirubin as variables related to liver function; triacylglycerol, total cholesterol, high density lipoprotein (HDL), low density lipoprotein (LDL), and very low density lipoprotein (VLDL) as variables related to dyslipidemia.

### 2.6. Statistical Analysis

The study subjects were divided into two groups: OBI/II (subjects with BMI between 30 and 39.99) and OBIII (subjects with with BMI ≥40) [21, 22]. The groups were compared using the generalized linear model (GzLM), taking into account the most suitable type of distribution for each dependent variable (gamma or Gaussian) and identity link function using the post hoc Bonferroni test. The choice of the best model was based on the lowest AIC (Akaike information criterion). To investigate the influence of BMI on the performance in cognitive tests, a linear regression test with a dichotomous predictor was used. Comparisons between independent variables (BMI) and ordinal dependent variables, such as education levels and degree of hepatic steatosis, were analyzed using the Mann–Whitney test. To verify the influence of the BMI on the iron parameters, a simple linear regression was performed, respecting the type of distribution of the variables. To verify the degree of the correlation among the variables of interest, the Pearson correlation test was performed for variables with normal distribution and the Spearman correlation test for nonnormal variables (considered statistically significant when *p* ≤ 0.05).

## 3. Results

**Figure 2:**
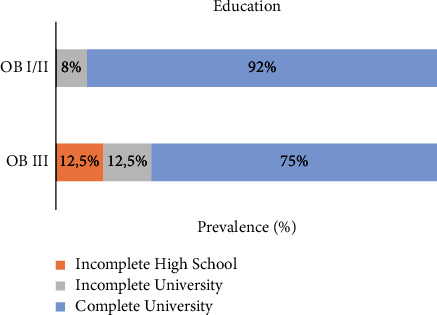
Education levels. Education levels were converted in ordinal variables and the Mann–Whitney test was applied, *P* > 0.05.

The study participants (female with obesity) were categorized according to their BMI: OB I/II (30 ≤ BMI < 40) or OBIII (40 ≤ BMI). Both groups presented similar age and education levels, but differed in BMI, body fat mass, visceral fat area normalized by height, and waist-to-hip ratio, as expected (Table 1 and Figure 2). Fat-free mass and skeletal muscle mass were not significantly different.

In order to verify whether the higher degree of obesity can negatively affect cognitive performance, a set of tests were applied, as shown in Table 2. OBIII presented lower scores in trial 1 and total recall of verbal paired-associate test. Linear regression analysis indicated that the degree of obesity has a negative impact on the performance in trial 1 and total recall of VPA test but not on the score of delayed recall (Table 3). In digit span, Toulouse–Pieron cancellation test, stroop color, and word test, both groups showed similar performance (Table 2). These data suggest that severe obesity can lead to poor performance in learning and memory processing compared to obesity I and II.

**Figure 3:**
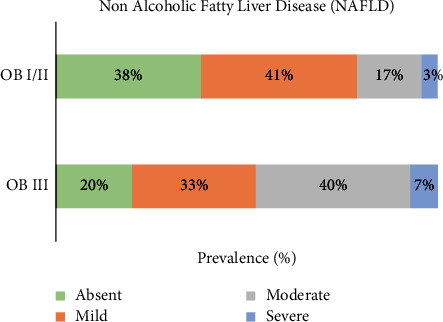
Prevalence of nonalcoholic fatty liver disease. Hepatic steatosis was graded according to ultrasound imaging. Distinct grade (mild, moderate, and severe) was converted to ordinal number and the Mann–Whitney test was performed (*p* > 0.05).

Parallel to cognitive tests, several metabolites and hormones were evaluated. Both groups showed very similar levels of indicators for glycaemia, dyslipidemia, thyroid hormone, and adipokines (Table 4). Although changes in blood glucose indicators have been associated with worse cognitive performance in general population, in our subjects with obesity, these indicators had no impact as covariates on the performance of the VPA test (Table 5). The OBIII group showed a higher percentage of subjects with moderate and severe hepatic steatosis based on ultrasound imaging, but this difference was not statistically significant (Figure 3). Furthermore, the groups showed the levels of biochemical markers related to liver diseases such as alanine aminotransferase and aspartate aminotransferase within the normal ranges, which are respectively 8–43 U/L and 7–45 U/L (Table 4).

**Figure 4:**
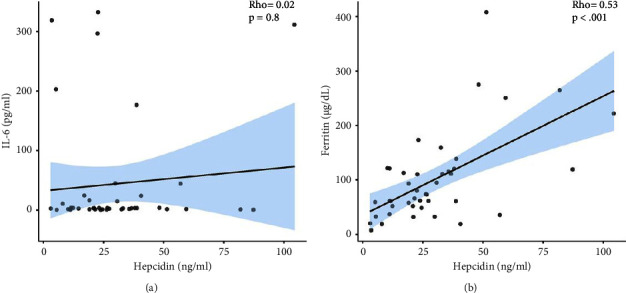
Correlations between hepcidin and IL-6 and ferritin. The Spearman correlation test was carried out. (a) IL-6 did not correlate with hepcidin (rho = 0.02; *p*=0.8), but (b) ferritin showed moderate positive correlation (rho = 0.53; *p* < 0.001). IL-6, *n* = 43; and hepcidin, *n* = 43.

Regarding iron status, total iron binding capacity (TIBC), degree of transferrin saturation (TSAT), hemoglobin, mean corpuscular volume (MCV), mean corpuscular hemoglobin (MCH), and red cell distribution width (RDW) were significantly different between groups, while folic acid and vit B12 were not (Table 6). Regression analysis indicated that BMI is negatively associated with serum iron and TSAT and positively associated with TIBC (Table 7). These results indicate that individuals with a higher degree of obesity are more prone to develop iron deficiency than OBI/II. Although several variables that indicate iron status were different between the groups, the levels of hepcidin and ferritin were similar in both groups (Table 6). The data from the literature suggest that hepcidin expression can be increased by IL-6 signaling [23], but we did not observe significant correlation between IL-6 and hepcidin (rho = 0.02 *p*=0.8) (Figure 4(a)). On the other hand, hepcidin levels showed significant positive correlation with ferritin levels (rho = 0.53 *p* < 0.001) (Figure 4(b)). Considering that both proteins can be enhanced by low grade inflammation, this correlation is somewhat expected [24]. The lack of the correlation between hepcidin and IL-6 has been also observed in other study [25], and further investigation is required to better understand the regulation of ferritin and hepcidin in obese population.

**Figure 5:**
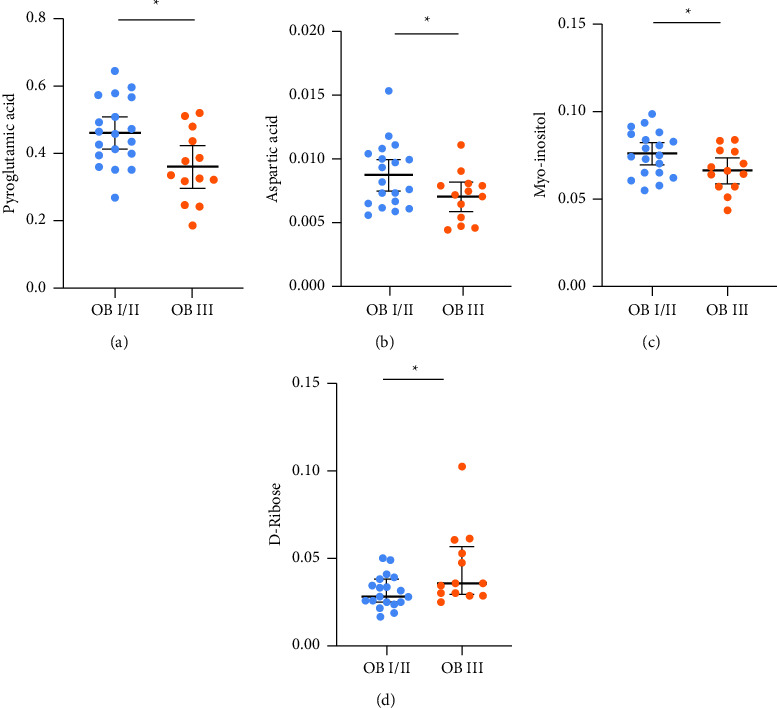
Relative abundance of metabolites between groups. The relative abundance of pyroglutamic acid (a), aspartic acid (b), myoinositol (c), and D-ribose (d) was compared between groups using GzLM analysis. Data are shown with respective mean and IC95 for normal distribution (a–c) or median and interquartile for variables that do not have a normal distribution (d). OB I/II (*n* = 19) and OB III (*n* = 13). ^*∗*^*p* < 0.05.

Iron is an essential micronutrient for oxygen transport and energy metabolism. Thus, in order to gain a deeper insight into the metabolic changes that occur in severe obesity, we also evaluated the plasma metabolites using an untargeted metabolic profiling strategy and GC-MS. For this analysis, we randomly selected 32 samples (19 OBI/II and 13 OBIII). In this analysis, 53 unique metabolites were identified, which were manually categorized according to their biochemical pathway (Figure 1). Considering that adipose and skeletal muscle tissues can significantly contribute to the profile of circulating metabolites, we verified the correlation between the levels of metabolites and BFM, VFA, and SMM. Figure 1 shows the correlation between BFM and the level of metabolites. Only 3 metabolites showed statistically significant correlation: 3-methyl-2-oxovaleric acid showed moderate negative correlation with BFM, while trans-hexa-dec-2-enoic acid and oleic acid showed moderate positive correlation with BFM and VFA (Table 8). None of the metabolites showed significant correlation with SMM. These data indicate that, in general, amino acids-related metabolites are reduced in OBIII compared to OBI/II, while metabolites related to fatty acids are increased. When analyzed by the group, 4 metabolites were significantly different between OBI/II and OBIII groups: levels of pyroglutamic acid, aspartic acid, and myoinositol were reduced in OBIII, and D-ribose was increased in OBIII (Figures 5(a)–5(d)).

## 4. Discussion

Obesity has been cited as one of the 12 disease-modifiable risk factors for dementia when prevented [26]. Because obesity is a complex pathological condition and each individual can be affected by a huge range of comorbidities; it is plausible to think that multiple mechanisms might be involved in the relationship between obesity and cognitive function. Considering that obesity is mainly characterized by metabolic disorders, and that healthy brain functions depend on adequate energetic balance, the investigation of metabolic profile with concomitant brain function is a compelling approach for the identification of risk factors and better understanding of the pathophysiological processes that lead to dementia [27].

In this sense, we hypothesized that the comparison between the groups of higher and lower degree of obesity can help to narrow down the potential mechanisms associated with cognitive impairment that happen in obesity. The rationale of this hypothesis is based on the facts that several metabolic adaptations might happen during the development of severe obesity from eutrophic states, and that not all the changes would be pathological [28]. On the other hand, because several comorbidities of obesity are known to aggravate with a higher degree of obesity [29], the comparison between the distinct degrees of obesity can be focused on finding these aggravating factors.

In our study, severe obesity showed worse performance in the VPA test compared to class I/II obesity. Although many previous studies reported that glucose toxicity and insulin resistance were factors that lead to the altered brain function [30–32], in our study, we did not observe the impact of these variables on the cognitive test, probably both OBI/II and OBIII groups showed similar levels of glycemia, glycosylated hemoglobin, insulin, and HOMA-IR. Thus, when we compare different classes of obesity groups, diabetes-related variables would no longer worsen the cognitive function. Instead, we observed that several markers of iron status were altered in the OBIII group compared to the OBI/II group. Iron deficiency has been associated with a poor cognitive performance in several types of population, including the patients with Alzheimer disease [33] and children [34]. Studies have shown that iron deficiency without anemia has a long-term impact on cognition affecting attention span, motor skills, and sensory perception [35]. Similarly, a study demonstrated that patients who have experienced iron deficiency in early life showed lower scores on tests of language, perception, and motor functions when compared to patients with normal iron status [34].

It is noteworthy that iron deficiency is known to be more prevalent in obesity than in the general population [36]. In a review article, the authors suggest that iron deficiency associated with obesity can worsen the cognitive performance in obese individuals [37]. Indeed, a recent article showed that obese children with lower serum iron levels had lower scores on cognitive tests and impaired academic performance [38]. Our data corroborate these findings, since OBIII presented reduced iron status and poorer performance in memory tests. It is also important to realize that our data do not oppose the previous findings about the effects of insulin resistance and glucose toxicity, since our subjects of the studies presented obesity and both groups did not differ in glycemia, HOMA-IR, and insulin levels.

With the adipose tissue expansion, the oxygen supply to the tissue can be impaired, which stimulates the infiltration of monocytes and macrophage and overproduction of chemoattractants and proinflammatory cytokines such as TNF-*α* and IL-6 [39]. IL-6 is a positive regulator of hepcidin via the JAK-STAT signaling pathway. Hepcidin, in turn, reduces the ferroportin levels and, consequently, the iron recycling and absorption, causing functional iron deficiency [40]. However, our data showed that neither IL-6 nor hepcidin levels were significantly different between OBI/II and OBIII, indicating that poor iron status observed in OBIII might be caused by other factors such as insufficient dietary iron intake.

Iron is an important cofactor for several enzymes and complexes that reside in the mitochondria, where most oxidative reactions of macronutrients occur. Thus, altered iron status in OBIII can impact the way a body produces its energy as well as the profile of circulating metabolites. In our study, we observed that higher degrees of obesity showed lower levels of pyroglutamic acid, myoinositol, and aspartic acid and higher levels of D-ribose compared to lower degrees of obesity. It is still unclear whether these metabolites were altered due to the altered iron homeostasis, and further studies are required.

Nonetheless, pyroglutamic acid appears to be indirectly associated with iron metabolism. Pyroglutamic acid is a metabolic by-product formed during the glutathione synthesis and it accumulates when the level of glutathione is low [41]. Glutathione plays important roles in iron metabolism and the biogenesis of Fe-S cluster, a prosthetic group present in many proteins involved in the oxidation of macronutrients such as electron transport chain complexes [42]. A previous study demonstrated that the activities of the enzymes involved in glutathione synthesis were increased in anemic erythrocyte, probably as a protective mechanism against oxidative stress caused by iron deficiency [43], and in this case, the level of pyroglutamic acid would be decreased. Based on these findings, it is possible to link reduced pyroglutamic acid in the OBIII group with the lower iron status.

Dietary iron deficiency also appears to increase the activity of the enzymes involved in pentose phosphate pathways (PPP) in erythrocytes in the rat model [44], which can then increase the D-ribose levels. In humans, the activity of glucose-6-phosphate dehydrogenase (G-6-PD), one of the enzymes of PPP, is required for the regeneration of reduced glutathione [45]. Thus, the increase of the G-6-PD activity can also be one of defense mechanisms against oxidative stress caused by iron deficiency, and as a consequence, it can cause the increase in pentose levels. D-ribose can easily surpass the brain barrier, and higher concentration of D-ribose can promote the production of advanced glycation end-products (AGE), which can trigger proinflammatory signaling in neural cells, impair the spatial memory function, and induce depressive behavior and anxiety in mice [10, 46]. Higher levels of D-ribose were also detected in urine samples of patients with Alzheimer disease [46].

**Figure 6:**
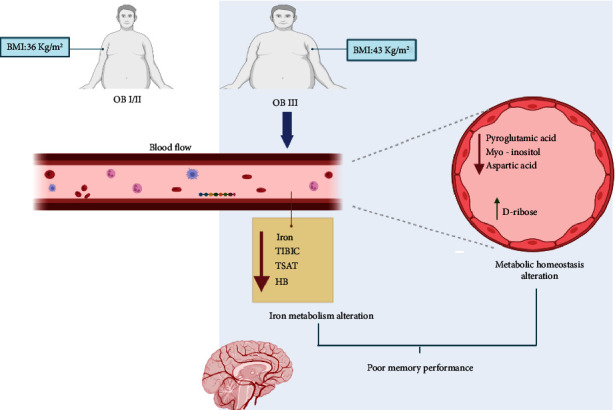
Subjects with class III obesity showed lower iron status, which may have altered the levels of some circulating metabolites related to oxidative stress and the cognitive performance.

In summary, our data showed that OBIII presented lower scores in the VPA test and lower iron status, which is known to impair cognitive function in the general population. Thus, a similar association between iron deficiency and cognitive function can occur in severe obesity, while a higher degree of obesity negatively affects the iron homeostasis. Regarding metabolic profiling, although not all the differences observed between OBI/II and OBIII groups can be mechanistically explained, based on our data and several previous studies, it is plausible to hypothesize that the reduction of pyroglutamic acid and increased D-ribose are the consequences of the oxidative stress generated by iron deficiency, and oxidative stress and consequent proinflammatory pathways could negatively impact brain function (Figure 6). Thus, possibly, better management of iron homeostasis in obesity can reduce the risk ratio of obesity for dementia and other comorbidities.

The primary limitation of this study is the small sample size. Also, the data about menstruation cycle and eutrophic group would be helpful to better interpret the iron status of participants. Nonetheless, because age is one of the main predicting factors for menopause, and both groups showed similar distribution of age, we can infer that perimenopause or menopause would not be the main factor that differentiates both groups in the cognitive function, even though previous studies have demonstrated that perimenopause may affect cognitive function [47, 48]. On the other hand, our findings add to the literature by showing the impact of obesity on cognitive function and possible involvement of iron deficiency.

## Figures and Tables

**Table 1:** Body composition of study participants.

	OBI/II (*n* = 29)	OBIII (*n* = 16)	*P* value
Age (years)	36.3 ± 7.04	35.7 ± 7.25	0.80
Body mass index (kg/m^2^)	36.3 ± 1.65	43.4 ± 3.21	<0.001
Body fat mass (kg)	45.9 ± 5.20	60.8 ± 6.44	<0.001
Body fat mass (%)	46.5 ± 3.95	52.1 ± 2.00	<0.001
Visceral fat area/height (cm^2^/m)	106 ± 11.0	138 ± 17.7	<0.001
Waist-to-hip ratio	0.96 ± 0.06	1.05 ± 0.05	<0.001
Fat-free mass (kg)	52.8 ± 5.48	55.8 ± 5.23	0.08
Skeletal muscle mass (kg)	29.5 ± 3.36	31.3 ± 3.03	0.07

Data are represented as mean with respective standard deviation. *P* values are results of the GzLM test. OBI/II, grade I and II obesity; OBIII, grade III obesity.

**Table 2:** Score of cognitive tests.

	OBI/II (*n* = 29)	OBIII (*n* = 16)	*P* value
*Verbal paired-associate test*			
Recall in trial 1 (*n*)	4.66 ± 1.49	3.31 ± 1.58	0.005
Total recall (*n*)	17.8 ± 3.36	14.6 ± 3.59	0.003
Delayed recall (*n*)	6.48 ± 1.35	5.81 ± 1.60	0.13

*Digit span*			
Forward (*n*)	7.11 ± 1.77	7.56 ± 2.76	0.50
Reverse (*n*)	5.86 ± 1.62	5.50 ± 1.83	0.49
Total score (*n*)	12.9 ± 2.37	13.1 ± 4.28	0.89

*Toulouse–Piéron cancellation test*			
Hit (*n*)	120 ± 32.6	103 ± 27.2	0.07
Failure (*n*)	13.8 ± 27.4	16.4 ± 23.5	0.74
Failure/hit (*n*)	0.15 ± 0.35	0.17 ± 0.25	0.73
Work efficiency (*n*)	106 ± 47.6	86.3 ± 39.2	0.16

*Stroop color and word test*			
Time 1 (seconds)	37.2 ± 5.59	40.5 ± 4.99	0.05
Time 2 (seconds)	45.3 ± 9.01	48.9 ± 9.19	0.20
Time 3 (seconds)	69.0 ± 17.6	73.8 ± 15.4	0.37

Data are represented as mean score with respective standard deviation. *P* values are results of the GzLM test. OBI/II, grade I and II obesity; OBIII, grade III obesity.

**Table 3:** Linear regression analysis between verbal paired-associate test and BMI.

	Estimate	SE	*R* ^2^	*P* value
Verbal paired-associate test				
Recall in trial 1 (*n*)	−1.34	0.47	0.15	0.007
Total recall (*n*)	−3.20	1.07	0.17	0.005
Delayed recall (*n*)	−0.67	0.45	0.04	0.14

Linear regression analysis is with BMI as the dichotomous predictor. The table shows the estimate (coefficient *β*), SE (standard error), *R*^2^, and *p* value.

**Table 4:** Biochemical data.

	OBI/II (*n* = 29)	OBIII (*n* = 16)	*P* value
Glycaemia (mg/dL)	97 ± 33	104 ± 31	0.50
Glycated hemoglobin (%)	5.54 ± 1.12	5.98 ± 0.99	0.20
Insulin (um/L)	22.9 ± 13.1	27.0 ± 34.9	0.57
C-peptide (ng/mL)	3.61 ± 1.85	3.81 ± 2.18	0.75
HOMA-IR (value)	5.69 ± 4.26	8.39 ± 15.2	0.35
Alanine aminotransferase (U/L)	28.8 ± 17.2	23.8 ± 7.22	0.23
Aspartate aminotransferase (U/L)	23.8 ± 12.2	18.3 ± 3.94	0.05
Gamma-glutamyl transferase (U/L)	32.4 ± 25.9	37.4 ± 14.4	0.48
Total bilirubin (mg/dL)	0.46 ± 0.18	0.38 ± 0.12	0.10
Direct bilirubin (mg/dL)	0.14 ± 0.05	0.11 ± 0.03	0.01
Indirect bilirubin (mg/dL)	0.32 ± 0.15	0.27 ± 0.11	0.32
Amilase (mg/dL)	63.3 ± 21.8	44.1 ± 13.0	<0.001
Triacylglycerol (mg/dL)	156 ± 132	113 ± 58.3	0.17
Total cholesterol (mg/dL)	199 ± 43.0	185 ± 32.1	0.27
HDL (mg/dL)	51.2 ± 11.0	52.2 ± 9.89	0.77
LDL (mg/dL)	120 ± 38.2	112 ± 31.1	0.45
VLDL (mg/dL)	27.7 ± 15.8	21.5 ± 8.42	0.11
TSH (*μ*UI/L)	2.85 ± 1.60	2.29 ± 1.43	0.23
T4 (ng/dL)	1.61 ± 2.17	1.03 ± 0.17	0.19
PCR (mg/dL)	3.47 ± 5.38	2.36 ± 2.75	0.50
IL-6 (pg/mL)	47.5 ± 104	37.7 ± 85.0	0.73
Leptin (ng/mL)	52.4 ± 30.0	53.5 ± 26.0	0.90
Adiponectin (*μ*g/mL)	3.73 ± 4.19	2.73 ± 1.45	0.31
FGF-21 (pg/mL)	368 ± 679	244 ± 391	0.56

Data are represented as mean with respective standard deviation. *P* values are results of the GzLM test. HOMA-IR, homeostasis model assessment of insulin resistance; HDL, high density lipoprotein; LDL, low density lipoprotein; VLDL, very low density lipoprotein; TSH, thyroid stimulating hormone; PCR, protein C reactive; FGF-21, fibroblast growth factor 21.

**Table 5:** Cognitive VPA test using blood glucose indicators as covariates.

	OBI/II (*n* = 29)	OBIII (*n* = 16)	*P* value
*Verbal paired-associate test*			
Trial 1 (*n*)	4.66 ± 1.49	3.31 ± 1.58	0.03

*Covariant*			
Glycaemia (mg/dL)	97 ± 33	104 ± 31	0.97
Glycated hemoglobin (%)	5.54 ± 1.12	5.98 ± 0.99	0.75
Insulin (um/L)	22.9 ± 13.1	27.0 ± 34.9	0.48
HOMA-IR (value)	5.69 ± 4.26	8.39 ± 15.2	0.42

Total recall	17.8 ± 3.36	14.6 ± 3.59	0.02

*Covariant*			
Glycaemia (mg/dL)	13.8 ± 27.4	16.4 ± 23.5	0.62
Glycated hemoglobin (%)	0.15 ± 0.35	0.17 ± 0.25	0.44
Insulin (um/L)	106 ± 47.6	86.3 ± 39.2	0.31
HOMA-IR (value)	5.69 ± 4.26	8.39 ± 15.2	0.35

Data are represented as mean with respective standard deviation. *P* values are results of the GzLM test.

**Table 6:** Iron status.

	OBI/II (*n* = 29)	OBIII (*n* = 16)	*P* value
Serum iron (*μ*g/dL)	79.8 ± 28.2	65.1 ± 20.8	0.06
TIBC (*μ*g/dL)	304 ± 34.9	336 ± 39.0	0.013
TSAT (%)	27.5 ± 10.0	18.6 ± 6.86	0.003
Ferritin (*μ*g/L)	110 ± 90.0	78.7 ± 55.7	0.15
Hepcidin (ng/mL)	28.7 ± 22.1	30.8 ± 23.0	0.77
Hemoglobin (g/dL)	14.0 ± 0.93	12.6 ± 2.73	0.017
Hematocrit (%)	41.9 ± 2.92	40.6 ± 2.33	0.14
MCV (fL)	88.0 ± 3.92	85.3 ± 4.95	0.047
MCH (g/dL)	29.3 ± 1.72	27.9 ± 1.96	0.014
MCHC (g/dL)	33.3 ± 1.10	32.7 ± 0.979	0.07
RDW (%)	13.0 ± 1.21	14.1 ± 1.09	0.005
Folic acid (ng/mL)	9.18 ± 3.24	8.99 ± 3.20	0.86
Vit B12 (ng/L)	505 ± 310	471 ± 170	0.68

Data are represented as mean with respective standard deviation. *P* values are results of the GzLM test. TIBIC, total iron binding capacity; TSAT, transferrin saturation; MCV, mean corpuscular volume; MCH, mean corpuscular hemoglobin; MCHC, mean corpuscular hemoglobin concentration; RDW, red cell distribution width.

**Table 7:** Linear regression analysis between iron marker and body mass index.

	Estimate	SE	Lower	Upper	*P* value
Serum iron (*μ*g/dL)	−1.96	0.93	−3.79	−0.130	0.042
TIBC	3.60	1.36	0.93	6.27	0.008
TSAT	−0.90	0.25	−1.36	−0.36	0.002
Ferritin (*μ*g/L)	−2.67	2.48	−7.46	4.94	0.28

Simple linear regression analysis with BMI as the predictor. The table shows the estimate (coefficient *β*), SE (standard error), IC95, and *p* value.

**Table 8:** Correlation between metabolites and body composition.

		BFM	VFA	SMM
3-Methyl-2-oxovaleric acid	*r*	−0.36	0.291	0.259
	*p*	0.047	0.106	0.152

trans-Hexa-dec-2-enoic acid	*r*	0.367	0.35	0.029
	*p*	0.039	0.049	0.876

Oleic acid	*r*	0.397	0.389	0.104
	*p*	0.025	0.028	0.571

Pearson correlation test was carried out. The table shows the correlation between body fat mass (BFM), visceral fat area (VFA), and skeletal muscle mass (SMM) with metabolites: 3-methyl-2-oxovaleric acid, trans-hexa-dec-2-enoic acid, and oleic acid. Pearson's coefficient *r* and the *P* values are shown in the table.

## Data Availability

The data supporting the findings of this study are available from the corresponding author upon reasonable request.

## Additional Points

(i) Class III obesity had worse impact on the memory-related cognitive test compared to class I and II obesity. (ii) Class III obesity was related to lower iron status compared to class I and II obesity. (iii) Changes in iron metabolism and circulating metabolites may have contributed to the poorer performance in memory-related cognitive tests.
